# Use of the Hashtag #DataSavesLives on Twitter: Exploratory and Thematic Analysis

**DOI:** 10.2196/38232

**Published:** 2022-11-15

**Authors:** Piotr Teodorowski, Sarah E Rodgers, Kate Fleming, Lucy Frith

**Affiliations:** 1 Department of Public Health, Policy & Systems University of Liverpool Liverpool United Kingdom; 2 NHS Digital Liverpool United Kingdom; 3 Centre for Social Ethics and Policy University of Manchester Manchester United Kingdom

**Keywords:** consumer involvement, patient participation, stakeholder participation, social media, public engagement, campaign, big data, research, trust, tweets, Twitter, perception, usage, users, data sharing, ethics, community, hashtag

## Abstract

**Background:**

“Data Saves Lives” is a public engagement campaign that highlights the benefits of big data research and aims to establish public trust for this emerging research area.

**Objective:**

This study explores how the hashtag #DataSavesLives is used on Twitter. We focused on the period when the UK government and its agencies adopted #DataSavesLives in an attempt to support their plans to set up a new database holding National Health Service (NHS) users’ medical data.

**Methods:**

Public tweets published between April 19 and July 15, 2021, using the hashtag #DataSavesLives were saved using NCapture for NVivo 12. All tweets were coded twice. First, each tweet was assigned a positive, neutral, or negative attitude toward the campaign. Second, inductive thematic analysis was conducted. The results of the thematic analysis were mapped under 3 models of public engagement: deficit, dialogue, and participatory.

**Results:**

Of 1026 unique tweets available for qualitative analysis, discussion around #DataSavesLives was largely positive (n=716, 69.8%) or neutral (n=276, 26.9%) toward the campaign with limited negative attitudes (n=34, 3.3%). Themes derived from the #DataSavesLives debate included ethical sharing, proactively engaging the public, coproducing knowledge with the public, harnessing potential, and gaining an understanding of big data research. The Twitter discourse was largely positive toward the campaign. The hashtag is predominantly used by similar-minded Twitter users to share information about big data projects and to spread positive messages about big data research when there are public controversies. The hashtag is generally used by organizations and people supportive of big data research. Tweet authors recognize that the public should be proactively engaged and involved in big data projects. The campaign remains UK centric. The results indicate that the communication around big data research is driven by the professional community and remains 1-way as members of the public rarely use the hashtag.

**Conclusions:**

The results demonstrate the potential of social media but draws attention to hashtag usage being generally confined to “Twitter bubbles”: groups of similar-minded Twitter users.

## Introduction

### Background

Well-established ways for sharing knowledge with the general public by researchers include academic publications, presentations, and media engagement (to name a few). However, previous research has raised concerns that the communication between scientists and the public needs to be more accessible and interactive than traditional engagement activities [1-3]. Public engagement, when it is a 2-way process of sharing, promoting, and disseminating research to the public [4,5], can improve trust between researchers and the public [6]. The growth of social media platforms, such as Twitter, a microblogging platform (up to 280 characters per post) [7], offers a more interactive way to engage with the public and can be particularly useful in promoting engagement around controversial topics. Twitter provides a less formal and more dynamic interaction among its users. Posts (tweets) are open to read for everyone, but only Twitter users can post (tweet) them (but Twitter is free and easy to sign up to). Users can reshare original tweets (retweet) with their audience (followers). Researchers are already active on Twitter to communicate their work as they can reach the public [8], colleagues in their field [9], policymakers, and practitioners [10].

One of the key issues in big data research and one subject to a prolonged public debate is the reuse of medical data for research. Often called big data, it has the potential to provide novel health solutions and improve health inequalities [11,12]. Non(re)use of data can negatively impact health services and research [13]. However, some public members are concerned about how their medical data are stored, controlled, (pseudo)anonymized, and reused [14,15]. Public trust and support are needed for big data projects to continue [16]. However, there remains little public understanding of big data research [14].

“Data Saves Lives” is a public engagement campaign that highlights the benefits of big data research, showing how patient data can be used securely to improve health care [17]. The campaign tries to build trust between researchers and the public. It was started by the University of Manchester's Health eResearch Centre in 2014. Since then, it has expanded outside the United Kingdom, and in 2019, it was launched in Europe. The Data Saves Lives European initiative is a multipartner project led by the European Patients’ Forum and the European Institute for Innovation through Health Data [18]. The campaign activities target social media, especially Twitter, using the hashtag #DataSavesLives. Hashtags allow the linkage of all posts on the same subject. Any user can use hashtags on Twitter, and to gain broad coverage, it is recommended to get as many Twitter users as possible to use the hashtag. However, this also means that the hashtag’s originators do not control by whom and how it is used. This can lead to highjacking of the hashtag by other users, who may use it for a different purpose than initially intended [19,20].

In 2021, the UK government and its agencies adopted the hashtag #DataSavesLives to support their plans to set up a new national database holding National Health Service (NHS) users’ medical data, which could be, in some circumstances, available for sharing with third parties [21]. The idea was driven by the COVID-19 pandemic and the recognition that data have the power to shape and improve health care services [22]. The plan was to collect 55 million patients' pseudonymized data in England to be reused (eg, to support services and research). This received heavy criticism from activists regarding lack of transparency around informed consent and confidentiality [23]. Patients would have only limited time to opt out of the scheme, and their consent was mentioned only once in the initial governmental policy documents [24]. The plan's legality was challenged, and there were concerns that medical professionals would refuse to comply by not sharing their patients' data [25]. Poor communication resulted in public concerns around this new scheme. British media outlets from the *Independent* to the *Daily Mail* described the plan as “controversial” [26,27]. These attitudes were not new, as a similar (but not linked) project was abandoned in the past due to negative public opinion [23,28]. Medical professionals had raised concerns about building trust with the public regarding new government plans. The British Medical Association and the Royal College of General Practitioners called for a better public engagement campaign to alleviate public fears [29]. One and half million people initially opted out of the scheme [30]. The government deferred the deadline for the public to opt out of the new database scheme due to public concerns [31]. Later, the policy was reviewed to discuss building trust with the public further [32]. The new governmental policy was published in June 2022 [33]. In contrast, there have been no such controversies in Europe or the adoption of #DataSavesLives by European public institutions.

Previous studies have explored public perceptions of big data research, but few have examined how online public engagement campaigns could promote the benefits of big data research. One paper discussed #DataSavesLives on Twitter, but its coverage was from September 2016 to August 2017 [34]. Our study expands on previous research and explores how the campaign’s hashtag was used when the UK government decided to adopt the hashtag in its campaign strategy. Thus, we cover the period of April-July 2021, when there was an ongoing discussion in news headlines around the newly proposed scheme.

### Models of Public Engagement

Science communication as a research area emerges from diverse fields and offers theoretical underpinnings for how researchers can engage with the public [3], where the public is understood as any person in society [35]. We use the terms “public” and “public members” in this paper as people who do not have a background in health care or big data research—laypeople. Three theoretical models of how researchers can engage with the public exist in the literature: deficit, dialogue, and participatory [36,37]. These differ in where they locate researchers or the public in the process of engagement [37].

#### Deficit Model

The deficit model is the oldest and nowadays heavily criticized model for being too passive a form of communication [35]. It is also known as the knowledge transmission model [38] as it assumes that the public has a limited understanding of the research, and through engagement, researchers can educate the public and explain the complexity of their work, promoting a researcher-centered model [2,39]. The model theorizes that if the public is not supportive of the ongoing research, researchers only need to explain it better to the public [39,40]. Thus, the underpinning problem is the public’s lack of understanding [3]. The weakness of this model is the ongoing need to educate the public, which can be only done through a top-down (and usually 1-way) approach, with researchers giving the public information and telling them how they should understand the issues. Empirical evidence has shown that the deficit model of engagement does not change public views toward science [41].

#### Dialogue Model

The dialogue model was developed in response to the mistrust the public had in research in general (but particularly in medical research) and the perceived failure and passivity of the deficit model to tackle that challenge successfully [40]. The public and researchers may have different perspectives and can interpret the same things differently [39]. The dialogue model recognises the need for an active exchange between researchers and the public, ensuring 2-way communication [37]. This communication can improve understanding among both groups as they can see different perspectives on the same issue. The dialogue model moves away from researcher-centredness in the communication process and invites public views on the research. Public understanding of science is no longer perceived as limited or inferior to researchers’ (as it was in the deficit model), but rather, it is perceived to offer a unique view. The model theorizes that the dialogue can further improve trust if researchers listen and implement public feedback. The public will not only understand the researchers’ perspectives better but also be more willing to act upon on their advice [42]. For example, it might be more willing to take a new medicine or participate in research.

#### Participatory Model

Shifting further the power balance between researcher and public, the participatory model argues for public-centredness in communication. Researchers and the public discuss the research agenda, and in contrast to the dialogue model, they also jointly find solutions. This democratization of the process has been argued to have the potential to improve the quality of information and reaching the public [43]. Both groups have something to gain from this cooperation [37]. In health research, it would come under the definition of public involvement, where work is being done together *with* the public rather than *for* it [44]. Growing research shows that public contributors (eg, lay members) are successfully involved in developing and shaping engagement of health care services [45].

### Research Questions

Underpinned by the (deficit, dialogue, and participatory) models of public engagement, this study aims to answer the following research questions:

- How was the hashtag #DataSavesLives used on Twitter as the UK government adapted the hastag in its campaign strategy?
- What were the attitudes toward the campaign among Twitter users using #DataSavesLives?

## Methods

### Data Collection

**Figure 1:**
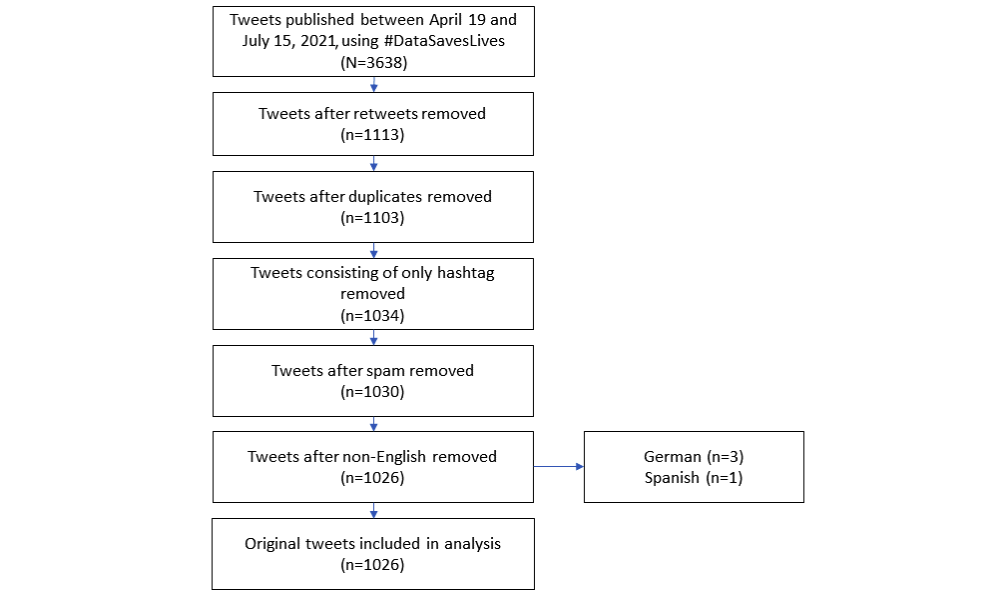
Process of cleaning data sets for qualitative analysis.

Tweets were recorded using NCapture software for Google Chrome. This web browser extension collects social media data, such as tweets (including retweets), and imports them directly to NVivo 12 (QSR International) for analysis. Only public tweets from the previous week could be recorded. NCapture does not guarantee that all tweets will be captured at once, as this depends on Twitter; thus, we captured tweets twice per week (Tuesday and Thursday) to get maximum coverage. If an individual tweet is captured twice, NVivo 12 uploads it into the data set only once. Tweets using the hashtag #DataSavesLives were captured for 3 months from April 27 to July 15, 2021. This covered tweets that were posted between April 19 and July 15, 2021. A total of 3638 tweets (including retweets) were collected. We cleaned the data set in NVivo 12 (see Figure 1). All retweets, duplicates, tweets consisting only of hastags, spam, and tweets in languages other than English were removed. After cleaning the data set, 1026 (28.2%) tweets were used in the qualitative analysis. Data saturation was deemed to have been reached. This assumption is based on previous research, which successfully conducted a qualitative analysis of fewer than 1000 tweets and provided novel insights into the online discussion through Twitter hashtags [46-48].

### Analysis

The analysis was conducted using NVivo 12. NCapture downloaded tweets as 1 data set to NVivo 12 software, and this enabled us to visualize the collected data data.

Descriptive statistics were used to summarise the top 40 user locations, the most active accounts, and the top hashtag used alongside #DataSavesLives and identify the most prominent tweet (based on the number of retweets). We included both tweets and retweets in this analysis to get a broader picture of all Twitter users using the hashtag.

To understand attitudes toward the campaign aims among Twitter users, each original tweet was manually assigned a category as having a positive, neutral, or negative attitude toward the campaign and big data research. The principles and techniques found in content analysis guided this process [49]. We jointly created a short description of each category and then conducted a pilot coding of a sample of tweets during the team meeting. Based on these discussions, an experienced Twitter researcher (author PT) systematically coded all remaining tweets.

Second, we undertook an inductive thematic analysis [50,51]. PT manually coded all tweets, and the team met to identify, review, and refine themes and choose the quotes representing them. Our research team is interdisciplinary, we work in and outside big data research, and 1 author (KF) is based outside the university, ensuring that we have both insider and outsider perspectives. Further analysis was carried out by mapping the thematic analysis results to the public engagement models, which offer insights into how Twitter users used the hashtag #DataSavesLives. Previous research has shown that the engagement techniques can be successfully mapped under these 3 engagement models [36].

### Ethical Considerations

The University of Liverpool Ethics Committee (approval no. 9815) granted ethical approval. All captured data are publicly available online. Following established practice [52,53], when we used a direct quote, authors (excluding organizations) were informed and given an option to opt out. No one asked to opt out, and 1 person requested a copy of the published paper. We did not include pictures, links, and emoticons.

## Results

### Descriptive Statistics

Of all tweets (N=3638) published in this period, the top 40 locations (excluding “unknown”) were from the United Kingdom, showing that the use of the hashtag is still mostly based in the United Kingdom. Other countries included the United States, Australia, Germany, Spain, and Belgium (see Table 1). The discussion was dominated by professionals. Of the 10 most active accounts using the hashtag (which represents n=1746, 48%, of all tweets), all were nonindividual accounts, such as organizations, networks, or public bodies. All public body accounts were linked to the UK’s NHS (see Table 2).

The most prominent tweet had 56 retweets, and it discussed a new webinar on big data research and concerns around data privacy. Some organizations, such as the Health Data Research UK, regularly promoted the benefits of big data research using the hashtag [54].

Most of the hashtags used alongside the campaign were neutral or positive. The top 10 included #healthdata (n=239, 65.8%), #covid19 (n=134, 3.7%), #nhs (n=102, 2.8%), #ai (n=101, 2.8%), #healtac2021 (n=91, 2.5%), #digitalhealth (n=89, 2.4%) #health (n=88, 2.4%), #testmining (n=84, 2.3%) #research (n=81, 2.2%), and #data (n=65, 1.8%). The negative anticampaign hashtag #DataGrab, which was used by Twitter users accusing the UK government of trying to sell their medical data, appeared 9 times in the whole data set and 5 times in original tweets, thus rarely appearing alongside #DataSavesLives, showing little cross-over between these 2 hashtags.

**Table 1:** Locations of Twitter users using #DataSavesLives (N=3638 tweets).

Country	Tweets, n
United Kingdom	2247
European Union (including Spain, Germany, and Belgium)	76
United States	56
Australia	44

**Table 2:** The 10 most active Twitter accounts using #DataSavesLives.

Twitter account	Tweets using #DataSavesLives, n (%)	Type of organization running the account
@hdr_uk	480 (13.2)	Nonprofit organization
@usemydata	353 (9.7)	Nonprofit organization
@nhsx	261 (7.2)	Public body
@nhsdigital	132 (3.6)	Public body
@datasaveslives	125 (3.4)	Nonprofit organization
@apha_analysts	97 (2.7)	Network
@uk_healtex	85 (2.3)	Network
@economics_unit	68 (1.9)	Public body
@medconfidential	66 (1.8)	Campaign group
@pioneer_hub	63 (1.7)	Nonprofit organization

### Attitudes

Discussion around #DataSavesLives was largely positive (n=716, 69.8%) or neutral (n=276, 26.9%) toward the campaign. There was some sarcasm in the negative attitudes (n=34, 3.3%) but no dark humor or personal attacks, which has been found in some other Twitter studies. This shows that the debate was generally conducted in a professional fashion, contrary to many politicized social media discussions [28,55,56].

Positive comments included reporting on successful, ongoing, or future projects that had benefitted the public when using big data.

The University is partnering with experts from across the UK to launch a £2m data hub for mental health. The hub promises to speed up research into mental health and improve inclusiveness for disadvantaged groups #MentalHealth #DataSavesLives

EdinburghUni

This evidence of public benefit can be seen in examples of how big data helped the response to the COVID-19 pandemic.

When the pandemic hit in 2020 we urgently looked at whether we could use routine data feeds to produce a more rapid cancer data set that would help quantify the impact of COVID-19 on cancer services. This is one example of how that work is now being used #DataSavesLives @PHE_uk https://t.co/4Eu1QgxXGm

EllissBrookes

Twitter users often emphasised how important or relevant was their work around big data research, thus linking it to the campaign’s underpinning rationale of showing that the reuse of medical data can change and even, indeed, save people’s lives.

Our Hubs are working to improve health data so that researchers & innovators are better able to use it to enable discoveries that improve people’s lives! #DataSavesLives Find out more: https://t.co/ZKQoaUWSos

HDR_UK

Often, organizations would quote stakeholders (eg, public members) to support these statements. There were calls for more public involvement and better data linkage.

Neutral tweets shared job opportunities, information about upcoming conferences, webinars, or new publications and asked people to participate in surveys or studies on big data research.

Hear from a super panel of speakers on Tues 25 May 10:00 -11:30 - A researcher’s journey to accessing patient data. #datasaveslives #admindata

SCADR_data

Negative tweets did not always take issue with the campaign itself but raised concerns about the lack of public trust in the opt-out deadline for the new UK database scheme. Others picked up on wording used in the hashtag and pointed out that the hashtag only appeals to professionals, not the public, and uses emotions to try to generate public support.

It's the wholly presumptuous nature of this scheme that is so abhorrent in my mind #DataSavesLives' the classic 'appeal to emotion' rolled out time and again as dogma in an attempt to upend logic #DataAsAsset is clearly much closer to reality

griffglen

### Thematic Analysis

**Figure 2:**
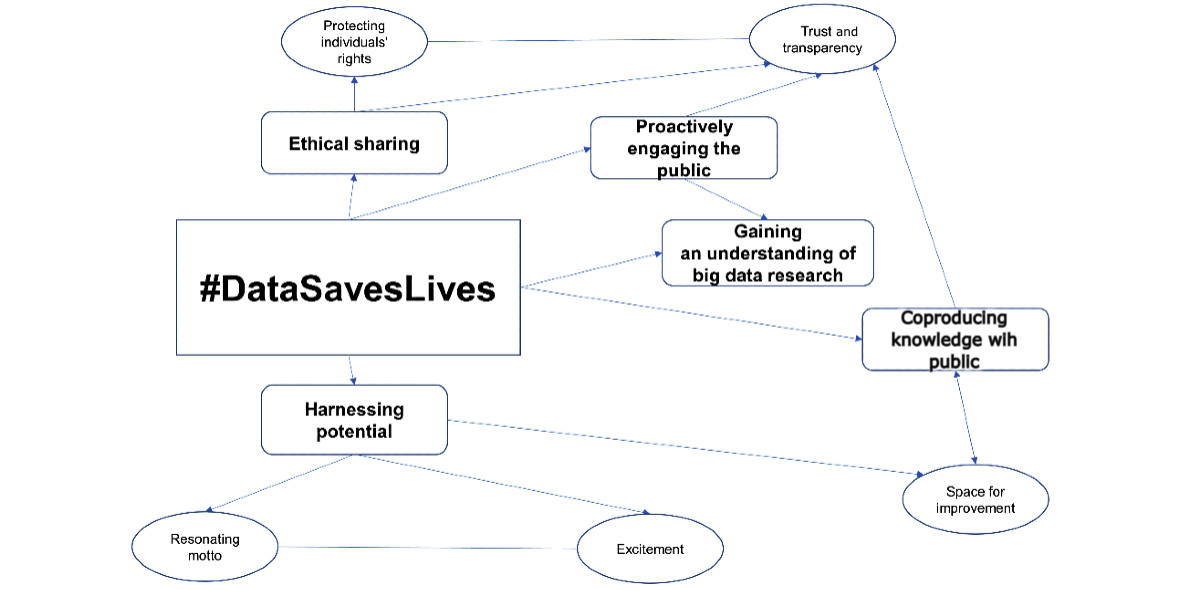
Key connectors and relationships between themes and subthemes.

We constructed 5 interlinked themes divided into 5 subthemes (Table 3) to illustrate how the debate around #DataSavesLives appears on Twitter. Figure 2 presents these key connectors and relationships between subthemes. We present the themes under the public engagement models of deficit, dialogue, and participatory.

**Table 3:** Themes and subthemes derived from the #DataSavesLives debate on Twitter through reflexive thematic analysis.

Themes	Subthemes
Ethical sharing	Trust and transparency Protecting individuals' rights
Proactively engaging the public	N/A^a^
Co-producing knowleadge with public	N/A
Harnessing potential	Excitement Space for improvement Resonating motto
Gaining an understanding of big data research	N/A

^a^N/A: not applicable.

#### Deficit Model

##### Harnessing the Potential

Tweet authors on the whole thought that big data has the potential to offer benefits to patients (eg, in the development of new medicines). Harnessing the potential of big data is visible in the following 3 subthemes: excitement, space for improvement, and resonating motto. The COVID-19 pandemic is present here but only as an additional argument for the claim that big data research is helpful for tackling new challenges.

##### Excitement

Tweet authors were often excited to announce new research projects and share study results (especially when showing how it has made some fundamental change or had the potential for real-life impact). Some of the tweets included authors who are passionate about the subject and others who were excited to participate in new studies. The researchers’ success was recognized and noticed by the broader research community (eg, receiving an award). Other tweets refer to upcoming events where authors were publicizing their next presentation (this refers to both single events or conferences).

This is one of the most exciting pilots I've seen up close. How we can link patient data, what the analysis tells us and how we can provide evidence to make change for patient benefit. #datasaveslives

SarahM_Research

##### Resonating Motto

Underpinning the campaign's motto is the argument that linking data and big data research saves and improves people's lives. This was a resonating motto, with many tweets about how the usage of medical data made an impact and provided new solutions. Tweets were either generic (relating to the benefits of big data research in general) or referred to specific research projects (both completed and ongoing).

'Data makes the unknown known' @margaretgrayson @useMYdata @NHSConfed #NHSReset #datasaveslives

ConyersRebecca

##### Space for Improvement

Tweets also argued for some changes to ensure the maximum benefit of big data. There were calls for more investment in big data research infrastructure, showing that big data research is still developing.

Predictive data modelling could lead to better humanitarian outcomes, but we are missing half the data needed. Time to act! #DataSavesLives.

Enovacom_en

Twitter users also recognized that some of these changes had to happen soon to offer more benefits from research.

Ahead of a crucial @G7, @NMRPerrin argues for the urgent need for better coordination across the global data sharing landscape https://t.co/aw8Apgw5Ku #datasaveslives @GS_Humphreys @royalsociety @GloPID_R

ICODA_research

##### Gaining an Understanding of Big Data Research

This theme is about reaching others (including the public but primarily other professionals, policymakers and researchers) and offering an opportunity to learn more about individual projects.

The hashtag offered an opportunity to call people to action, to apply for job openings (mostly research related), and to welcome new team members. Some tweets asked other researchers to support big data research or answer ongoing consultations or surveys.

Only a few days left to apply for this! Working with a great team enabling the #HealthData infrastructure to support #COVID19 #research. Secondments welcome, remote working too so location flexible. #HealthData #DataSavesLives

LaraEdw001

This illustrates how the hashtag was used among similar-minded people to publicize new opportunities and events.

Tweets also allow readers to learn more about big data projects, attend events, follow online chats, and read recent blogs or papers. This is mostly passive and focused on dissemination rather than engaging.

Check out this thread from @HDR_UK with examples of how #DataSavesLives being added throughout June

NIHRresearch

#### Dialogue Model

##### Ethical Sharing

The need for ethical, safe, and lawful sharing of data in big data research and the importance of doing it right were a prominent theme in the data. Two subthemes deal with key aspects of achieving these aims: protecting individuals' rights, and trust and transparency.

##### Protecting Individuals' Rights

There is agreement that big data research offers new opportunities for innovation. However, the impact on individual rights remains the main concern. This was particularly around how the data are used, who has access, how secure it is, and whether patients could be identified. Many organizations attempt to reassure people by telling them that any data usage is secure and transparent.

There were concerns that health data could be sold to private companies to make a profit. Some tweets linked that concern with people's decisions to opt out in the United Kingdom from using their medical data for research. Some admitted that the public has not been properly or sufficiently engaged around and about these issues.

@Axelheitmueller, you're completely correct, the benefits of data sharing are immense for the health of our nation. For some reason there's a narrative that we intend to make a profit from data. This is simply not the case. We do not, and we will not sell data! #datasaveslives

simonrbolton

##### Trust and Transparency

Associated with individual rights are trust and transparency, which underpin public support for big data research. Tweet authors argued that public trust is essential for big data research to succeed and that the processes of data sharing have to be transparent and follow well-established principles. Otherwise, it risks undermining public support as the public will lose confidence. There have been comments within the UK context that recent political events have undermined that trust, which is also shown by the hashtag #DataGrab. Trust and transparency are perceived as the building blocks of successful research projects and are often the rationale that underpins public engagement.

Sharing my data can aid research needed to improve health care for myself and others with chronic illness. However, there does need to be clearer reassurance that data won't be misused so that individuals can make an informed choice. #GPDPR #nhsdataoptout #DataGrab #datasaveslives

LucindaH19

##### Engaging the Public

There was a push in the tweets to have better engagement with the public and encourage conversations about big data research. Some approaches to this included avoiding jargon and ensuring that events are free to attend. There was also some media engagement as Twitter users shared links where researchers took part in media interviews. In addition, media outlets were tagged as Twitter users tried to catch their attention. These engagement activities are intended to help the public understand the value of big data research better. However, if they limited themselves to only explaining big data research to the public, they could be seen as following a deficit model of engagement, with its associated limitations.

Health data research can be confusing sometimes and full of buzzwords and jargon. This article clearly explains how health data is used and why it's so important. If you donate your data to health research you could help improve future health care. #DataSavesLives #DataScience

genscot

#### Participatory Model

##### Coproducing Knowledge With the Public

Public contributors could be successfully involved in big data research. These are public members who actively contribute to research projects, ensuring that research is conducted *with* and not *to* or *about* them. Views on how much the public should be involved differed. Some tweets explore the active role of the public in studies as public contributors, whereas others focus only on reaching people and showing them the benefits of big data research (as shown in the previous theme, proactively engaging the public).

Tweets refer to involving public members in big data projects. In this theme, there is a call for more public involvement. Tweet authors showed examples of how involving the public as active contributors had a positive impact on their research.

There were calls for more public control, thanking patients for sharing their medical data for research (not opting out), and recruitment calls for new public contributors in big data projects.

None of this would be possible without our Data Trust Committee – the diverse and inclusive group of patients and members of the public, who review every data access request and make decisions based on the Five Safes and, ultimately, the public's best interest. #datasaveslives

useMYdata

It is also important to involve patients in developing registries or data collections. Also citizens, because they produce the data and therefore, as owner of the data, they should have a seat on the “Datatable” too. #patientsinvolved #datasaveslives #MTF2021

Birgitpower

## Discussion

### Principal Findings

This study explored how #DataSavesLives was used on Twitter. The findings clearly show that the debate was mostly positive toward the campaign. This is not surprising as most participants were organizations, academics, and institutions that work in big data research. Our findings confirm previous research on the #DataSavesLives hashtag—that it is being used to identify similar-minded projects around big data and to spread positive messages toward big data research, particularly when there are public controversies [34].

We mapped the results of our thematic analysis into models of public engagement. This showed that the largest number of themes were within the deficit and dialogue models and only 1 theme was included in the participatory model. Each model has its uses, and a hierarchy is not necessarily the most useful way to understand them [37]. The public engagement campaign can be placed within all of these models [39]. However, if the campaign wants to improve trust with public members, more active exchange with the public is needed. This can be achieved by moving more campaign-related activities into activities that would conform with the dialogue or participatory models. One way of doing this is to engage more Twitter users to participate in active discussion online. Previous research has shown that Twitter can accommodate a vibrant debate around challenging topics [57]. How Twitter users used the hashtag #DataSavesLives is not a new phenomenon in Twitter discussions about science. For example, a study that explored science festivals found that organizations mostly focus on distributing information and only a smaller part of the Twitter activity is actually interactive [58].

The hashtag usage remains limited to similar-minded Twitter users—a Twitter bubble. The results indicate that communication around big data research is driven by the professional community and research remains 1-way because the public rarely uses the hashtag. This confirms previous research showing that government science organizations do not fully use the potential of social media to engage with the public [59]. Within this data set, there was only a limited appearance of negative hashtags, such as #DataGrab (n=5), which was used during the UK debate on the new database scheme. This elicits questions about how successful the campaign is in achieving its goals of engaging with the public. The campaign messages do not target any seldom-heard communities but rather focus on researchers and professionals. Twitter bubbles are not a new phenomenon, and Sunstein [60] describes them as an “echo chamber” that amplifies the already existing beliefs of Twitter. However, despite public members not using the hashtag themselves, it does not exclude the possibility that they are exposed to these messages, as research [61] has shown that researchers with over 1000 followers on Twitter have diverse followers (eg, media representatives and public members). The #DataSavesLives campaign shares many aspects of 1-way communication and remains in the deficit engagement model. However, many engagement campaigns have limited interaction with the public at the beginning but can improve over time [39]. Thus, based on previous research, the campaign has potential to develop.

The campaign was relaunched in Europe in 2019, but there were only 4 Tweets in languages other than English. Our findings indicate that the campaign remains UK centric as the most active Twitter accounts are based in the United Kingdom. The high activity of the government-run UK organizations poses the question whether the hashtag and campaign could continue on Twitter without their involvement. The use of #DataSavesLives remains limited on Twitter. However, this can be explained by the type of messages published online. Most were positive or neutral toward the campaign, whereas the negative emotions on social media spread faster than the positive emotions [62]. This should not encourage Tweet authors to start appealing to negative emotions but rather recognize the limitations of the positive engagement campaign.

Ethical challenges and issues of trust and transparency around big data research remain a concern for the public [63]. In 2014, NHS England launched a promotional campaign showing how medical records would become part of a larger database. The project called Care.data was controversial, and a previous study explored the #caredata controversy on Twitter [28]. At that time, there was a distinct lack of public engagement or involvement in big data projects. There now seems to be a clear recognition that the public should be proactively engaged and involved in discussions about big data projects. There is an improvement in how professionals and organizations perceive public involvment. According to Tweet authors, the public can be involved at various points. Some suggest only explaining the benefits of big data research, while others call for and present examples of having public contributors involved in research (eg, governance). Limited the public understanding of the use of big data remains 1 of the largest challenges [64], and more engagement could, arguably, improve this situation.

Based on our research findings, PT participated in a Tweet chat hosted by the European Patients’ Forum as part of their regular conversations around big data research on Twitter. We hoped that this would allow more online engagement within the dialogue model. The discussion considered the online movement and how social media is spreading the campaign's message [65]. We found it beneficial to present our research, discuss the emerging findings, and engage with Twitter users who had used the hashtag #DataSavesLives. This was an opportunity to talk to the people involved in running the campaign about what they thought the future of the campaign might be. The public member contributing to the discussion pointed out the need for more actively involving the public around big data research. This further confirmed our findings and the need for researchers to shift engagment to dialogue and participatory models.

### Limitations

Organizations in the United Kingdom were the main authors of downloaded Tweets. This limits our understanding of how much the results of our study reflect public attitudes toward the campaign and questions whether the public is actually aware of it. Twitter offers limited demographics about its users. Some data, such as location, were unknown (eg, online location appeared as the third-most popular location, used by 7.6% of Twitter users) or included 2 or more countries. In addition, because some demographic data were unavailable, we cannot say whether the usage differs among different age groups or other attributes.

The activity of an automated Twitter account, a bot, can influence Twitter traffic. A bot aims to create tweets and retweets to expand the coverage of their messages. We manually coded the data set and did not notice this kind of activity, but this does not guarantee that it was not there.

Data collection took place when there were new database scheme controversies in the United Kingdom, which could have influenced some traffic and messages. Future research should check whether the Twitter discussion has shifted depending on the context. Our study explored only usage of #DataSavesLives in English, but it is also available in German as #DatenrettenLeben. Our study focused on Twitter, the main microblogging platform, where users often discuss contentious or political topics. However, the hashtag is also available on other social media (Facebook and Instagram), and future research could explore whether engagement there differs from Twitter. Other research could also focus on negative hahstags toward sharing routinely collected health data, such as the already mentioned #DataGrab.

### Conclusion

This study shows how Twitter users used #DataSavesLives when the hashtag was adopted by the UK government and during the UK domestic controversies around data linkage and sharing. There are growing expectations from funders that researchers will engage with the public. Social media campaigns, such as #DataSavesLives, may offer an opportunity to further this goal. This study expands our understanding of the #DataSavesLives campaign. The results demonstrate the potential of social media and recognizes the need for engaging with a wider range of opinions and different Twitter constituencies. Thus, researchers need to identify new ways of actively engaging a wider range of the general public. There is a need to move engagement activities from a deficit model to dialogue and participatory models that include active 2-way engagment between researchers and public members and genuinely include the public in meaningful involvement. Future research could explore whether and how Facebook and Instagram users use the hashtag.

## Abbreviations

NHS: National Health Service.
